# Impact of Anaemia on Functional Status in Patients with Chronic Heart Failure

**DOI:** 10.14789/ejmj.JMJ25-0051-OA

**Published:** 2026-01-27

**Authors:** SHAHREEN KHALID, ALI UMER, SYEDA UMM E ABEEHA ZAIDI, MUHAMMAD SAJAWAL SALEEM, ABDUL SAMAD ABBASI, SHAUMILE HASAN KHAN, BHAVNA SINGLA, SHIVAM SINGLA, SUNITA KUMAWAT, FOLASADE FALAJU, SAADIA KANWAL

**Affiliations:** 1 Department of Community Medicine, Bakhtawar Amin Medical and Dental College, Multan, Pakistan; 1 Department of Community Medicine, Bakhtawar Amin Medical and Dental College, Multan, Pakistan; 2 Department of Acute Medicine, Doncaster Royal Infirmary, Doncaster and Bassetlaw Teaching Hospitals, NHS Foundation trust, Doncaster, South Yorkshire, UK; 2 Department of Acute Medicine, Doncaster Royal Infirmary, Doncaster and Bassetlaw Teaching Hospitals, NHS Foundation trust, Doncaster, South Yorkshire, UK; 3 Department of Medicine, Islamabad Medical and Dental College, Islamabad, Pakistan; 3 Department of Medicine, Islamabad Medical and Dental College, Islamabad, Pakistan; 4 Department of Internal Medicine, Indus Hospital & Health Network, Karachi, Pakistan; 4 Department of Internal Medicine, Indus Hospital & Health Network, Karachi, Pakistan; 5 Department of Medicine, Liaquat University of Medical and Health Sciences, Jamshoro, Pakistan; 5 Department of Medicine, Liaquat University of Medical and Health Sciences, Jamshoro, Pakistan; 6 Department of Internal Medicine, Fatima Memorial Hospital Medical College, Lahore, Pakistan; 6 Department of Internal Medicine, Fatima Memorial Hospital Medical College, Lahore, Pakistan; 7 Department of Internal Medicine, Erie County Medical Center, Buffalo, NY, USA; 7 Department of Internal Medicine, Erie County Medical Center, Buffalo, NY, USA; 8 Department of Internal Medicine, Tidal Health Peninsula Regional, Salisbury, MD, USA; 8 Department of Internal Medicine, Tidal Health Peninsula Regional, Salisbury, MD, USA; 9 Department of Internal Medicine, PWC/St. Francis Medical Center, Lynwood, California, USA; 9 Department of Internal Medicine, PWC/St. Francis Medical Center, Lynwood, California, USA; 10 Department of Internal Medicine, Thumbay University Hospital, Ajman, United Arab Emirates; 10 Department of Internal Medicine, Thumbay University Hospital, Ajman, United Arab Emirates; 11 Department of Medicine, Shalamar Hospital, Lahore, Pakistan; 11 Department of Medicine, Shalamar Hospital, Lahore, Pakistan

**Keywords:** anaemia, chronic heart failure, functional status, Kansas City Cardiomyopathy Questionnaire, Pakistan

## Abstract

**Objectives:**

Chronic heart failure (CHF) restricts daily functioning and quality of life. Anaemia is frequent in CHF and can impair functional outcomes. This research evaluated the effectiveness of anaemia on functional status among Pakistani CHF patients.

**Materials and Methods:**

This cross-sectional study comprised 310 patients aged 18 years and above who attended outpatient clinics and community health centres in Islamabad and Rawalpindi. The structured questionnaire was used to gather data on demographics, self-reported anaemia, and medical history. Functional status was assessed using the Kansas City Cardiomyopathy Questionnaire (KCCQ-12). Descriptive statistics, chi-square, t-tests, correlation, two-way ANOVA, and multiple regression were analysed.

**Results:**

239 respondents (77%) were anaemic. The KCCQ-12 scores (30.9 ± 3.9) were lower in anaemic patients (34.1 ± 3.2), and there was an inverse relationship between functional status and anaemia (r = -0.338, p < 0.001). There were significant effects of anaemia (p < 0.001), age (p < 0.001), and both (p < 0.001) in two-way ANOVA, with older anaemic patients more impaired. Regression revealed that anaemia (B = -2.10), age (B = -1.25), female gender (B = -0.95), comorbidities (B = -0.48), and smoking (B = -0.65) were the predictors of poor functional status (p = 0.05).

**Conclusions:**

Anaemia is ubiquitous among CHF patients in Pakistan and closely associated with diminished functional capacity, especially in the elderly. Anaemia screening and treatment should be part of CHF management to enhance the quality of life.

## Introduction

Chronic heart failure (CHF) is a rising critical health issue and a prominent cause of hospitalisation and healthcare expenses, especially among the elderly, where diastolic dysfunction is the leading cause, and prevalence ranges 1-2% which increases with older populations^1, 2)^. CHF is a clinical syndrome characterised by fatigue, dyspnea, and congestion. It is associated with the heart's inability to fill or eject blood effectively, leading to insufficient tissue perfusion and fluid retention^3)^.

Patients with CHF experience a gradual decline in functional ability, contributing to a significantly decreased QOL in physical, social, and emotional aspects^4, 5)^. Anaemia is a frequent coexisting condition in heart failure (HF) that is linked to poorer long-term prognosis and increased myocardial workload, and develops due to multifactorial mechanisms, including iron deficiency, underlying chronic inflammation, impaired erythropoietin activity, and neurohormonal activity^6, 7)^.

Multiple studies have reported a link between anaemia and increased short-term and long-term mortality; therefore, correcting anaemia before discharge is necessary to improve patient outcomes^8)^. Anaemia was detected in 15% of patients with congestive HF and was more common in advanced classes of NYHA, with 19% of patients in class III-IV experiencing anaemia as compared to 8% of patients in class I-II. There was no difference in haemoglobin levels, but anaemia was associated with a progressive decline in functional status and disease severity^9)^.

Anaemic individuals with HF have higher in-hospital mortality, cardiovascular complications, hospitalisation, and healthcare expenditure relative to non-anaemic patients^10)^. A reduced hematocrit is associated with higher 1-year mortality and a higher likelihood of rehospitalisation, and a 1% decrease in hematocrit is associated with a 2% increase in mortality^11)^.

The connection between functional impairment and anaemia in CHF is a crucial factor that can be utilised to enhance patient management and outcomes. The research will assess how anaemia affects functional status in patients with CHF, and also provide the researcher with a reference point for possible therapeutic interventions that can improve QOL and reduce the disease burden.

### Rationale

Chronic heart failure is a growing health issue in Pakistan, as the rising prevalence rates of cardiovascular disease and the lack of access to specialised care present a significant burden on patients and healthcare systems. In this regard, most patients exhibited lower functional capacity, which affects their ability to perform daily tasks and lead a satisfactory life. These patients usually experience anaemia, which is not taken seriously in the normal course of clinical practice, but which can also complicate such symptoms as fatigue and dyspnea. The interaction between anaemia and CHF in the local population may inform the identification of high-risk groups and the targeting of regions where specific interventions could enhance patients' day-to-day functioning.

Although its clinical significance is not well reported, the effect of anaemia on functional status among CHF patients in Pakistan is not well reported. Factors potentially influencing the prevalence of anaemia and its impact on heart failure outcomes could include region-specific cultural, dietary, and healthcare-related factors. The research can further inform local healthcare providers by focusing on this population and educating them on how to manage their affairs better. Early assessment of functional status and anaemia can aid clinicians in directing interventions that target not only correcting laboratory abnormalities but also enabling patients to enhance their well-being and perform their normal daily activities.

### General objective

To find out the impact of anaemia on the functional status of CHF patients in Pakistan.

### Specific objectives

• To assess the prevalence of anaemia in patients suffering from CHF.

• To evaluate the functional capacity of patients with CHF by standard measurements.

• To determine possible demographic or clinical characteristics that can affect the impact of anaemia on functional status.

## Materials and Methods

### Research design and methodology

This was a cross-sectional study to describe the effects of anaemia on functional status in patients with chronic HF aged 18 years and over. The research was conducted in Pakistan, specifically in Islamabad and Rawalpindi, among patients with HF. The sample was obtained through the consecutive recruitment of participants from public and private facilities, including cardiology outpatient clinics (private) and community health centres (public). Therefore, it is heterogeneous in terms of age, gender, and socioeconomic level.

Data collection was performed using a formatted, self-administered questionnaire. In the analysis, participants reported demographic characteristics, comorbidities and self-reported anaemia. Functional status was measured using a standardised patient- reported measure that assessed the impact on daily activity, symptom burden, and general physical function. The use of this questionnaire-based approach facilitated elucidation of the association between reported anaemia and functional impairment, thereby improving understanding of the impact of anaemia on daily living and overall HRQoL in these patients.

### Sample size

An infinite population was used because the total number of adults with CHF and anaemia in the source population is not exactly known. The equation employed to calculate the sample size was:

\[n = \frac{Z^2 \cdot p (1 - p)}{d^2}\]

Where Z = standard score level of confidence desired, p = estimated proportion, and d = acceptable margin of error. In the present study, Z was set at 1.96 for a 95% CI, d at 0.05, and p at 0.50 (maximum variance) to maximise the sample size and account for the absence of local prevalence data. The sample size was estimated based on the assumption that 385 participants were needed^12)^.

During data collection, 311 respondents who met the eligibility criteria completed questionnaires and were included in the final analysis. A convenience sampling technique was employed in both public and private health facilities of Islamabad and Rawalpindi, as well as in cardiology outpatient departments (OPDs) and community health posts (CHPs). It should be noted that, because convenience sampling was employed, the results of this study may not be fully generalizable to the broader population of patients with CHF. Only those who met the study eligibility criteria and agreed to participate during the data collection period were included.

### Selection criteria

The individuals included were adults aged 18 years and above with a diagnosis of CHF, capable of comprehending and answering the questionnaire, and who provided informed consent to participate in the study. People who had either acute heart failure or had been in the hospital because of cardiac-related events in the last three months were not included. Patients with known haematological diseases that were not related to chronic heart failure, patients who are not able to complete the questionnaire because of medical impairment, language barrier, or other severe conditions, were also locked out, as they might confound the measurement of anaemia and functional status.

### Procedure and instruments

A structured questionnaire, completed by participants themselves, was used to collect the data. The instruments were completed by the respondents themselves in the outpatient clinics and community health centres of Islamabad and Rawalpindi, including both public and private cardiology clinics. There was availability of trained personnel to provide verbal instructions to participants who had difficulty comprehending any item due to literacy or language difficulties, without affecting their responses. Informed consent was obtained in writing from all participants before their participation.

### Demographic information

The first section of the questionnaire was designed to collect demographic data to describe the study sample and to investigate the potential correlation between anaemia and functional status. There were age, gender, marital status, education level, occupation, chronic diseases and lifestyle factors such as smoking. Additionally, respondents were asked a self-reported question about anaemia: Have you ever been told by a doctor or other medical professional that you had anaemia (low blood count)? This allowed it to distinguish as respondents those who reported a history of anaemia.

### Kansas City Cardiomyopathy Questionnaire (KCCQ)

The Kansas City Cardiomyopathy Questionnaire (KCCQ-12) was used in the present study to assess patients' perceived QOL relative to HF symptoms. The KCCQ-12 was created in 2000 by Dr John A. Spertus and colleagues at the Mid-America Heart Institute and consists of four domains: physical limitation, symptom frequency, QOL, and social restriction. The questionnaires are scored on a scale from 0 to 100, with higher scores indicating better health. The individual domain scores are then combined to create a unified score. The KCCQ-12 is a brief self-report tool that exhibits high internal consistency (Cronbach's α = 0.89). It can therefore also be used to assess the impact of heart failure symptoms on patients' daily functioning and well-being. This tool enabled a systematic, objective description of functional status in relation to self-reported anaemia among the subjects investigated^13)^.

### Data evaluation

The data analysis was conducted using IBM SPSS Statistics version 26. Categorical variables are presented as frequencies and percentages, whereas continuous variables are expressed as means and standard deviations. Independent-samples t-tests and one-way ANOVAs were used to analyse participants' functional status scores across demographic and clinical groups. Comparisons involving the categorical variables and their associations with effect size were performed using chi-square tests (effect sizes are reported as Cramer's V). In contrast, a two-way ANOVA was conducted to investigate the main effects of anaemia status, age, and interaction on functional status (KCCQ-12) scores. Point-biserial correlation was used to examine the association between anaemia status and KCCQ-12 scores. Predictors of functional status (anaemia and organic diseases, age, sex, and smoking) were also assessed by multiple linear regressions. All p-value tests were two-sided, and a p < 0.05 indicated statistical significance.

### Ethical compliance

This research was conducted in accordance with the ethical principles for human subjects, including the rights of individuals, their privacy, and protection. The Institutional Review Board of Islamabad Medical and Dental College approved the study (IRB #0307-25).

All participants were aware of the study's aim, procedures, and potential risks and benefits. All participants gave written consent. Participation was strictly voluntary, and individuals were informed that they were free to leave the study at any time without incurring any adverse consequences. All personal information was stored securely and used solely for research purposes, ensuring confidentiality. Missing or incomplete data were handled with care during the analysis. The pairwise deletion method was used to maximise data use, and questionnaires with response rates below 20% were omitted to ensure the validity and reliability of the findings. These measures demonstrated the integrity of the study and safeguarded the privacy and rights of all participants.

## Results

### Demographic characteristics of participants

Table 1 outlines the demographic features of the study participants (N = 310). Most participants were 65 years and above (150, 48%), followed by those aged 50-64 years (90, 29%), 35-49 years (40, 13%), and 18-34 years (30, 10%). Females (190, 61%) outnumbered males (120, 39%). Approximately half of the participants (149, 48%) were single, 87 (28%) were married, and 74 (24%) were divorced, widowed, or separated. In terms of education, the majority of them had secondary/matric education (108, 35%), intermediate/high school (68, 22%), a lesser percentage had no formal education (32, 10%), primary education (58, 19%), bachelor's degree (26, 8%), and post graduate education (18, 6%). Regarding residence, 132 (43%) resided in rural areas, 127 (41%) in semi-urban/town areas, and 51 (16%) in urban areas. Smoking status was nearly balanced, 141 (45%) former smokers, 139 (45%) current smokers, and 30 (10%) never smokers. The most prevalent medical conditions were diabetes mellitus (98, 32%), hypertension (87, 28%), chronic kidney disease (65, 21%), coronary artery disease/heart attack (33, 10%), heart failure (16, 5%), and COPD/asthma (11, 4%). In general, a significant percentage of participants were anaemic (239, 77%), and only 71 (23%) were non-anaemic.

**Table 1:** Demographic characteristics of participants (N = 310)

Variable	f (N)	%		Variable	f (N)	%
Age				Place of residence		
18-34 years	30	10		Urban	51	16
35-49 years	40	13		Semi-urban/Town	127	41
50-64 years	90	29		Rural/Village	132	43
≥ 65 years	150	48		Smoking status		
Gender				Never smoked	30	10
Male	120	39		Former smoker	141	45
Female	190	61		Current smoker	139	45
Marital status				Medical condition		
Single	149	48		Hypertension	87	28
Married	87	28		Diabetes mellitus	98	32
Divorced/Widowed/Separated	74	24		Chronic kidney disease	65	21
Educational level				Coronary artery disease/Heart attack	33	10
No formal education	32	10		Heart failure	16	5
Primary	58	19		COPD/Asthma	11	4
Secondary/Matric	108	35		Anemia status		
Intermediate/High school	68	22		Yes (anemic group)	239	77
Bachelor's degree	26	8		No (non-anemic group)	71	23
Postgraduate (master's or above)	18	6				

Note. f = frequency, % = percentage; Values are presented as N (%), N = 310; No statistical comparisons were performed for demographic variables in this table

### Correlation between anaemia status and functional status

Table 2 presents the point-biserial correlation between anaemia status and functional status (KCCQ-12 scores). There was a strong inverse association between anaemia status and KCCQ-12 scores (r = -0.338, t(308) = -6.30, p < 0.001, N = 310), indicating that participants with anaemia had significantly lower functional status than those without.

**Table 2:** Point-biserial correlation between anemia status (Yes/No) and functional status (KCCQ-12) score (N = 310)

Variables	1	2
Anemia status	-	r= -0.338, t(308)= -6.30, p= <0.001**
KCCQ-12	-	-

Note. Values represent Pearson correlation coefficients (r) between continuous variables; N = 310; ** = p < 0.001 was considered statistically significant and is denoted with double asterisk (**).

### Samples t-test comparing functional status

Table 3 presents the results of the independent- samples t-test for functional status between anaemic and non-anaemic patients. There was a significant difference in the mean KCCQ-12 score between the two groups (M = 30.9, SD = 3.9, N = 239; 77 vs. M = 34.1, SD = 3.2, N = 71; 23), t (308) = -6.31, p = 0.001. The 95% confidence interval for the mean difference (-4.20 to -2.20) and the effect size (Cohen's d = -0.85) were large, indicating that anaemia was significantly associated with worse functional status.

**Table 3:** Independent samples t-test comparing functional status (KCCQ-12 Scores) between anemic and Non-anemic patients (N = 310)

Variable	Yes (Anemic group) (N=239;77%) M±S.D	No (Non-anemic group) (N=71;23%) M±S.D	t	p	Cl 95%		Cohen's D
					LL	UL	
Kansas city cardiomyopathy questionnaire (KCCQ-12)	30.9 ± 3.9	34.1 ± 3.2	−6.31	<0.001***	-4.20	-2.20	-0.85

Note. Values are presented as Mean ± Standard Deviation; Independent samples t-tests were conducted to compare participants of both with and without Anemia groups; Group sizes are shown as N (%); Reported statistics include p-values, t-values, 95% Confidence Intervals (CI), and effect sizes (Cohen's d); A p-value < 0.05 was considered statistically significant, N = 310.

### Functional status by anaemia and age group

Table 4 presents the average KCCQ-12 scores by anaemia status and age group. The functional status of anaemic patients (N = 239) decreased with age, with the mean functional status highest in the 18-34 years group (M = 34.5, SD = 2.8) and the lowest in the 65 years and above group (M = 28.5, SD = 3.8). The same age-related reduction was observed in the non-anaemic group (N = 71), whose scores were consistently higher across all age groups, ranging from 36.0 ± 2.6 (18-34 years) to 32.8 ± 3.2 (65+ years). In general, the non-anaemic group (M = 34.1, SD = 3.2) reported better functional status than the anaemic group (M = 30.9, SD = 3.9). Among anaemic patients, the worst outcomes were reported in those with an age of ≥ 65.

**Table 4:** Means and standard deviations of KCCQ-12 scores by anemia status and age group (N = 310)

Anemia status	Age group	N	M ± SD
Anemic	18-34 years	20	34.5 ± 2.8
	35-49 years	25	33.0 ± 3.0
	50-64 years	70	31.2 ± 3.5
	≥ 65 years	124	28.5 ± 3.8
Total		239	30.9 ± 3.9
Non-anemic	18-34 years	10	36.0 ± 2.6
	35-49 years	15	35.0 ± 2.9
	50-64 years	20	34.0 ± 3.1
	≥ 65 years	26	32.8 ± 3.2
Total		71	34.1 ± 3.2
Overall		310	31.9 ± 3.9

Note. Values are reported as means ± standard deviations (M ± SD). Higher KCCQ-12 scores reflect better functional status; Anemic patients, particularly those aged ≥ 65 years, reported the lowest functional status; N = 310

### Age-related trends in functional status among anaemic and non-anaemic patients

Table 5 presents the outcomes of a two-way ANOVA examining the effects of anaemia status and age group on functional status (KCCQ-12 scores). The main effects of both anemia status (F (1, 302) = 17.46, p < 0.001, η 2 p = 0.055) and age group (F(3, 302) = 9.49, p < 0.001, η 2 p = 0.086) were significant, which shown that anemic patients were less functional and that the age group had significant main effects. Notably, the interaction between anaemia and age was also substantial (F(3, 302) = 6.72, p < 0.001, η^2^p = 0.063), indicating that the adverse effect of anaemia on functional status was more pronounced in the older age groups.

**Table 5:** Two-way ANOVA results for the effects of anemia status and age on KCCQ-12 scores (N = 310)

Source	Type III SS	df	MS	F	p	η^2^p
Corrected model	720.40	7	102.91	9.45	<0.001**	0.180
Intercept	31900.55	1	31900.6	2928.1	<0.001**	0.906
Anemia status	190.25	1	190.25	17.46	<0.001**	0.055
Age group	310.60	3	103.53	9.49	<0.001**	0.086
Anemia × Age	219.55	3	73.18	6.72	<0.001**	0.063
Error	3260.12	302	10.79	-	-	-
Total	319013.0	310	-	-	-	-
Corrected total	3980.52	309	-	-	-	-

Note. Dependent variable = Kansas City Cardiomyopathy Questionnaire (KCCQ-12); Significant main effects of anemia status and age group were observed, as well as a significant anemia × age interaction; Effect sizes are reported as partial eta squared (η^2^p); **= p < 0.001 considered significant

### Predictors of functional status in chronic heart failure patients

Table 6 presents the findings of the multiple linear regression analysis, which predicts functional status (KCCQ-12 scores) based on anaemia status, age, gender, medical condition, and smoking status. The general model was significant, and several predictors were significant as well. Strong negative predictors included anaemia status (B = -2.10, β = -0.225, p < 0.001) and age (B = -1.25, β = -0.200, p < 0.001), indicating that anaemia and age were associated with lower functional status. The effect of gender also differed, as females reported lower scores (B = -0.95, 8 = -0.105, p = 0.025). Also, medical conditions (B = -0.48, 2 = -0.160, p = 0.001) and smoking (B = -0.65, 2 = -0.130, p = 0.002) were significant negative predictors. Collectively, all these results suggest that anaemia, advanced age, gender (female), comorbid conditions, and smoking are significant predictors of worse functional status in patients.

**Table 6:** Multiple linear regression predicting Kansas City Cardiomyopathy Questionnaire (KCCQ-12) scores from anaemia status, age, gender, medical condition, and smoking status (N = 310)

Predictor	B	SE	β	t	p	95% CI LL	95% CI UL
Constant (Kansas City Cardiomyopathy Questionnaire) (KCCQ-12)	42.500	2.950	-	14.41	<0.001**	36.70	48.30
Anemia status	-2.100	0.410	-0.225	-5.12	<0.001**	-2.91	-1.29
Age	-1.250	0.310	-0.200	-4.03	<0.001**	-1.86	-0.64
Gender (Female)	-0.950	0.420	-0.105	-2.26	0.025*	-1.78	-0.12
Medical condition	-0.480	0.140	-0.160	-3.43	0.001**	-0.76	-0.20
Smoking status	-0.650	0.210	-0.130	-3.10	0.002**	-1.06	-0.24

Note. Multiple linear regression was conducted to identify predictors of Functional status (KCCQ012) Scores; Values include unstandardized coefficients (B), 95% confidence intervals (CI), standard error (SE), standardized beta coefficients (β), and p-values; *= p < 0.05, **= p < 0.01, ***= p < 0.001was considered statistically significant, N = 310.

### Graphical representation of predictors affecting functional status

**Figure 1:**
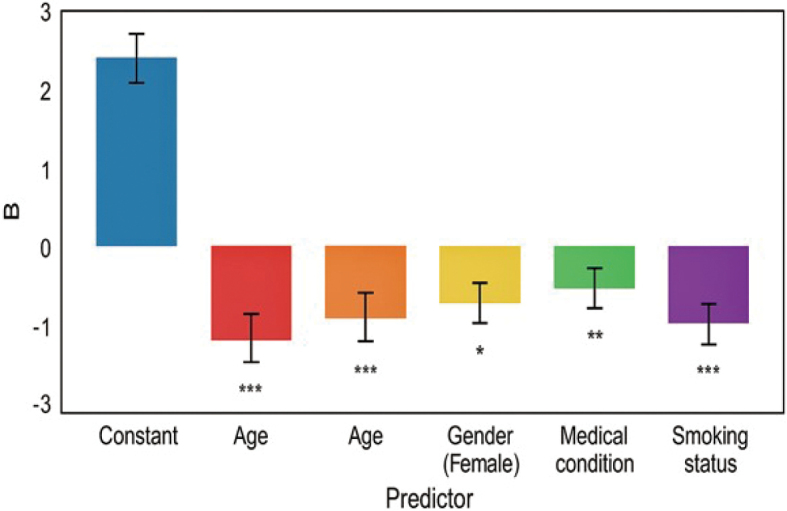
Bar chart of multiple linear regression predicting KCCQ-12 scores from anaemia status, age, gender, medical condition, and smoking status (N = 310)

Figure 1 presents the outcomes of a multiple linear regression analysis examining the relationship between KCCQ-12 scores and anaemia status, age, gender, medical condition, and smoking status in patients with CHF (N = 310). The intercept was positive, indicating a constant level of functional status. There were significant negative relationships between the KCCQ-12 scores and age, gender as females, and the presence of medical conditions and smoking status, which implies worse functional status in these populations. In particular, the most significant adverse effects were recorded by smoking status and age (p < 0.001), medical conditions (p < 0.01), and gender (female) (p < 0.05). These findings show that advanced age, gender, presence of comorbid medical conditions, and smoking are some of the essential factors predicting poor functional status among this group of patients.

### Gender differences in anaemia prevalence

Table 7 indicates the relationship between gender and anaemia status of patients with chronic heart failure. The percentage of males anaemic (169, 80%) was greater than that of non anaemic (41, 20%), and the rate of female anaemic (70, 70%) was less than that of non anaemic (30, 30%). The chi-square test revealed a statistically significant relationship between gender and anaemia status, χ2(1, N = 310) = 4.36, p = 0.037, with a small effect size (Cramer V = 0.12). This means that the proportion of males with anaemia was higher than that of females in this patient category.

**Table 7:** Comparison of gender and anemia status in chronic heart failure patients (N = 310)

Gender	Anemic, N (%)	Non-anemic, N (%)	Total, N (%)	χ^2^ (df = 1)	p	Cramer's V
Male	169 (80%)	41 (20%)	210 (68%)	-	-	-
Female	70 (70%)	30 (30%)	100 (32%)	-	-	-
Total	239 (77%)	71 (23%)	310 (100%)	4.36	0.037*	0.12

Note. Data are presented as N (%); the Chi-square test was used to assess the relationship between missed injuries and gender frequency; Statistical significance was considered at p < 0.001

## Discussion

This study investigated the impact of anaemia on the functional status of patients with CHF using the KCCQ-12. In our study, anaemia was strongly associated with worse heart-related functional status, as indicated by lower KCCQ-12 scores. This is in line with prior research on patients with advanced heart failure, where low haemoglobin was associated with more severe symptoms, decreased functional capacity, and lower survival^14)^.

Patients with anaemia had significantly lower KCCQ-12 scores than non-anaemic patients in our study, suggesting worse functional status. Consistent with previous findings, a study reported that anaemia in patients with heart failure with reduced ejection fraction was a strong predictor of worse quality of life^15)^. Older anaemic patients (> 65 years) had the poorest functional status, and the high interaction between anaemia and age points to older patients being specifically susceptible to the adverse effects of anaemia. The findings are corroborated by studies that have established anaemia as a potent predictor of physical disability among elderly heart failure patients^16)^.

Regression analysis confirmed that anaemia was also the independent predictor of reduced functional status, thus reinforcing previous observations on its negative impact on quality of life^14, 15)^. Older age was also a predictor of lower KCCQ-12 scores, consistent with prior evidence that older patients with HF are at higher risk for functional impairments^16)^. Females in our study had lower KCCQ-12 scores (i.e., worse functional status) than males. This is consistent with previous investigations showing women suffering from heart failure experience increased symptom burden and worse QOL in comparison to men^17)^. In addition, a higher prevalence of comorbidities was associated with lower KCCQ-12 scores, consistent with previous research suggesting that a heavy burden of comorbidities results in a stepwise decrease in functional status and exercise capacity^18)^. In our study, smokers also had lower KCCQ-12 scores compared with non- smokers, indicating worse functional status. This is consistent with other past reports that current smokers with heart failure report a health status much worse than never smokers and ex-smokers^19)^.

We also showed that men were slightly more likely to be anaemic than women, and the gender-anaemia association was weak but significant. This is in agreement with earlier studies, which have shown that sex and the impact of the presence of anaemia on clinical outcomes among chronic heart failure patients are influenced by gender^20)^.

### Clinical implications

The relationship shown here between anaemia and poor functional status highlights the importance of early intervention. Iron supplementation, erythropoietin-stimulating agents, and treatment of the underlying causes (e.g., chronic kidney disease or nutritional deficiencies) might alleviate symptoms, encourage activity and potentially reduce the number of hospital readmissions.

### Study limitations

Considering some shortcomings is necessary. To begin with, the research employed a cross-sectional design, which does not allow for the determination of cause-and-effect relationships between functional impairment and anaemia. Second, the status of anaemia was determined on self-report rather than by laboratory-confirmed haemoglobin levels, which could lead to recall bias or misclassification. Third, convenience sampling makes it challenging to generalise the results to the general CHF population in Pakistan. Additionally, unmeasured confounders, including medication use, dietary patterns, and socioeconomic status, may have influenced the observed results. Lastly, functional status was assessed using a patient-reported questionnaire, which, although validated, is prone to response bias.

### Recommendations

Longitudinal designs should be employed in future research to establish causality and determine the long-term impact of treating anaemia on outcomes such as hospital admissions, mortality, and quality of life. The inclusion of objective laboratory data on anaemia, such as haemoglobin, ferritin, and transferrin saturation, would improve classification and enable exploration of iron-deficiency anaemia as a distinct subgroup. Generalizability would be enhanced by expanding recruitment to multiple provinces in Pakistan, allowing comparisons across regions. Interventional studies evaluating the cost- effective management of anaemia in CHF patients, particularly in resource-limited settings, are warranted. Additionally, the interplay between anaemia, psychosocial factors, and health literacy can be examined to gain a more holistic understanding of how anaemia affects patient well-being.

## Conclusion

To summarise, this study has shown that anaemia is prevalent among patients with CHF in Pakistan, and it has a strong relation with poor functional status, especially in older patients and individuals with comorbidities. These results underscore the need to detect and manage anaemia earlier as part of the global approach to CHF. The treatment of anaemia may improve people's daily well-being and also reduce their overall perception of QOL, potentially even diminishing the need for hospitalisation. It could thus be exciting to include anaemia screening as standard in CHF management so that the outcomes of this at-risk subpopulation can be optimised.

## Author contributions

SS and AU conceived and designed the study and supervised the overall project. SUAEZ and MSS contributed to conceptual support, data interpretation, and critical revision of the manuscript. ASA and SHK were responsible for data analysis, manuscript preparation, and final approval. BS, SS, and SK contributed to the literature review and referencing. FF and BS assisted with data validation and statistical review. SK and SS contributed to formatting, proofreading, and preparation of tables and figures. All authors read and approved the final manuscript.

## Conflicts of interest statement

The authors declare that there are no conflicts of interest.
